# Endoscopic management of massive mercury ingestion: A case report

**DOI:** 10.1097/MD.0000000000006937

**Published:** 2017-06-02

**Authors:** Levente Zag, Gábor Berkes, Irma F Takács, Attila Szepes, István Szabó

**Affiliations:** a Emergency Department; b Department of Gastroenterology, Bács-Kiskun County Hospital and Teaching Hospital of University of Szeged, Kecskemét, Hungary.

**Keywords:** emergency medicine, endoscopy, mercury

## Abstract

**Rationale:**

Ingestion of a massive amount of metallic mercury was thought to be harmless until the last century. After that, in a number of cases, mercury ingestion has been associated with appendicitis, impaired liver function, memory deficits, aspiration leading to pneumonitis and acute renal failure. Treatment includes gastric lavage, giving laxatives and chelating agents, but rapid removal of metallic mercury with gastroscopy has not been used.

**Patient concerns:**

An 18-year-old man was admitted to our emergency department after drinking 1000 g of metallic mercury as a suicide attempt.

**Diagnosis:**

Except from mild umbilical tenderness, he had no other symptoms. Radiography showed a metallic density in the area of the stomach.

**Intervention:**

Gastroscopy was performed to remove the mercury. One large pool and several small droplets of mercury were removed from the stomach.

**Outcomes:**

Blood and urine mercury levels of the patient remained low during hospitalization. No symptoms of mercury intoxication developed during the follow-up period.

**Lessons:**

Massive mercury ingestion may cause several symptoms, which can be prevented with prompt treatment. We used endoscopy to remove the mercury, which shortened the exposure time and minimized the risk of aspiration. This is the first case where endoscopy was used for the management of mercury ingestion.

## 1 Introduction

Massive elemental mercury ingestion is rare in the literature. Swallowing metallic mercury is thought to be harmless because of poor absorption via the gastrointestinal tract^[1]^; however, prolonged exposure can cause systemic toxicity.^[2]^ In some cases, hepatic dysfunction^[3]^ and appendicitis^[4]^ have developed. Oral intake can be accompanied by aspiration, which complicates the outcome.^[5,6]^ Management is mainly conservative, including gastric lavage, administration of osmotic laxatives, and chelating agents.^[3,4,6–8]^^.^

Here, we report a unique case of massive elemental mercury ingestion, wherein the mercury was removed from the gastrointestinal tract by esophagogastroduodenoscopy (EGD).

## 2 Case report

An 18-year-old white boy was admitted to our emergency department because of drinking 1000 g of elemental mercury 3 hours earlier as a suicide attempt. His medical history included psychiatric hospitalization for schizotypal disorder. He denied alcohol and drug consumption. On admission, he complained of abdominal pain (3 on the visual analogue pain scale). Vital signs were normal, and the physical examination disclosed umbilical tenderness.

Laboratory findings were normal, except from slightly elevated aspartate aminotransferase (89 U/L) and alanine aminotransferase (193 U/L) values. These elevated values of unknown origin corresponded with previous laboratory findings in the past year. Blood and urine mercury levels were measured on the third day after exposure, and were 5 μg/L (normal <10 μg/l) and 6.2 μg/L (normal <5 μg/L), respectively.

**Figure 1:**
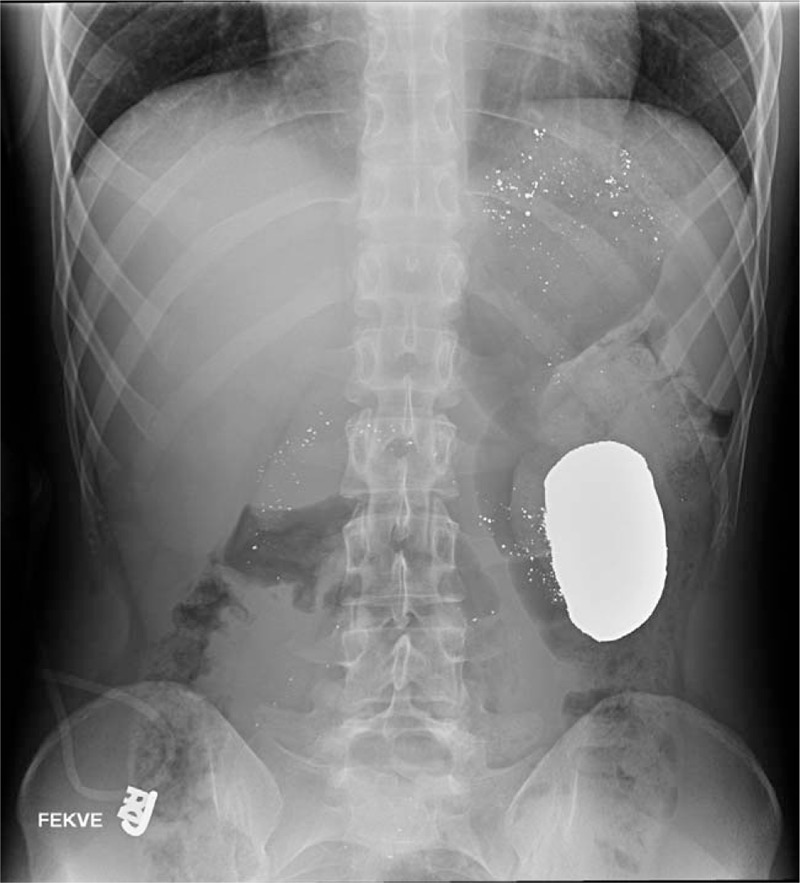
Metallic density in the area of the stomach. The mercury was ingested 3 hours before radiography. Anteroposterior radiography in supine position.

The abdominal radiograph revealed a metallic density in the region of the stomach or the jejunum on the left side of the abdomen (Fig. 1). After consultation with the National Toxicology Information Center, we decided to remove the ingested mercury from the gastrointestinal tract. The possibility of surgical removal was ruled out owing to the risk of mercury entering the peritoneal cavity. Furthermore, suction via gastroscopy or the use of laxatives for the facilitation of emptying was considered. Based on the radiograph, the most likely location of the mercury was the stomach. Given several reports of successful management of foreign body ingestion with endoscopy, an attempt at endoscopic removal was chosen. The patient was kept in a left prone position to prevent spontaneous gastric emptying.

**Figure 2:**
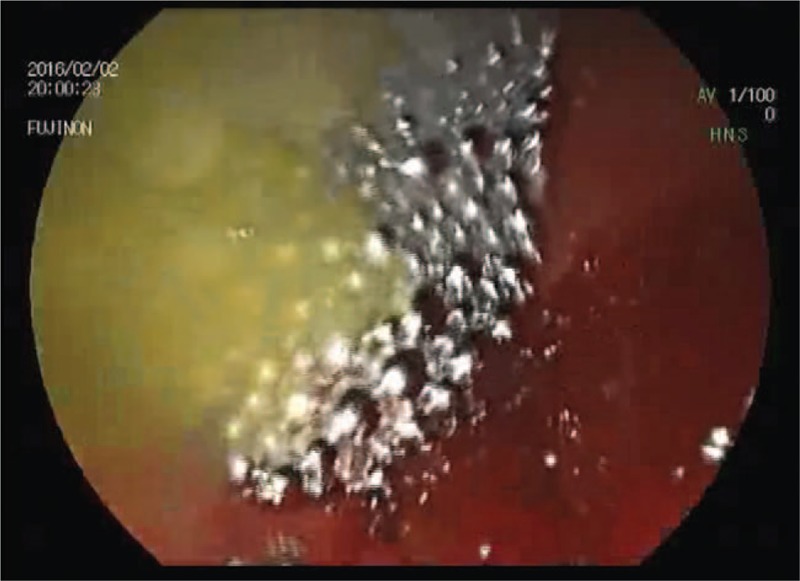
Droplets of metallic mercury in the stomach during endoscopy.

The patient was prepared for EGD with rapid sequence induction and intratracheal narcosis to prevent aspiration. During EGD, one large pool of mercury and several small mercury droplets were detected in the stomach and the duodenum. As a diagnostic endoscope was used, its relatively thin working channel (2.8 mm) did not allow for the successful desufflation of the pool of elemental mercury from the gastric fundus. Therefore, we decided to change the instrument to a duodenoscope with a significantly larger working channel (3.8 mm). EGD was performed under fluoroscopy to control the remaining droplets of mercury around the endoscope. With this endoscopic retrograde cholangiopancreatography (ERCP)-like combination of procedures, we were able to remove almost the entire amount of mercury during a 30-minute intervention (Fig. 2). A minimal amount of the mercury remained in the intestinal tract because food debris interfered with complete removal. A follow-up radiograph showed scattered punctate opacities in the gastric fundus and in the descending part of the duodenum.

After observation for a few hours in the intensive care unit, the patient was transferred to psychiatry. During a 3-month follow-up period, no symptoms of mercury intoxication were noted.

## 3 Discussion

In cases with an intact gastrointestinal system and passage, the absorption of metallic mercury is negligible.^[1]^ Nevertheless, several complications have been associated with metallic mercury ingestion. While passing through the colon, mercury can accumulate in the appendix and cause appendicitis.^[7]^ In one case, a mercury-filled tube ruptured in a patient after a right colectomy, and the mercury entered the retroperitoneum. The elemental mercury oxidized to toxic, absorbable ions, causing tremors and enterocolitis, which led to death.^[9]^ Oral exposure has been found to cause liver function impairment with jaundice 6 months later in 1 patient.^[3]^ Prolonged oral intake of elemental mercury caused nausea, lethargy, tremor, and an elevated urine mercury level in a man who had been eating 3 tablespoons of mercury for 12 weeks.^[2]^ Aspiration associated with vomiting or gastric lavage can complicate mercury ingestion. Neurological dysfunction, renal failure, and liver function impairment may occur because of the absorption of metallic vapors in the lungs.^[1]^ After metallic mercury ingestion, vomiting developed and led to aspiration in a man, causing severe pneumonitis and acute renal failure.^[5]^ Gastric lavage was applied to remove ingested mercury in a woman, but this provoked vomiting and aspiration. No pneumonitis developed, but the patient demonstrated impaired memory and disturbances in verbal-logical thinking.^[6]^ Abdominal discomfort, periumbilical pain, aphtoid ulcers, gum hyperemia, and swelling after vomiting may occur after ingesting a massive amount of metallic mercury.^[3,8]^ Blood and urine mercury levels can become elevated, and urine mercury levels may reach the toxic range (>50 μg/L).^[3,8]^

Based on the aforementioned clinical cases, rapid removal of mercury is needed to minimize detrimental effects on health. Depending on the duration of exposure, gastric lavage may be used, but this involves the risk of aspiration.^[6]^ Whole-bowel irrigation can be performed with osmotic laxatives.^[6,8]^ Grimes presented a case wherein the ingested mercury was removed with colonoscopy, when the mercury was found in the appendix.^[10]^ In our case, the mercury was in the stomach, which raised the possibility of endoscopic removal, considering the high efficiency of using endoscopy in removal of foreign bodies, including magnets, batteries, cement, or bezoars containing drugs from the stomach.^[11–13]^ Therefore, suction of the mercury by gastroduodenoscopy under endotracheal narcosis was chosen. The use of endoscopy prevented aspiration associated with gastric lavage, and the complications of surgical intervention were also avoided. The blood and urine mercury levels in this patient remained low, so the possibility of systemic intoxication was excluded. Endoscopy proved to be a safe method for clearing metallic mercury from the upper gastrointestinal system. EGD also has its limitations. If mercury passes through the duodenum, it cannot be removed from the lower intestinal regions with upper endoscopy. In case of the mercury scattered in the colon, bowel irrigation or colonoscopy may help to reduce the exposure time. Allergy to drugs used under general anesthesia is also a limiting factor.

In this article, we report on a patient who ingested a large amount of metallic mercury as a suicide attempt. Endoscopy has been previously used in numerous cases to remove foreign body from the stomach; however, liquid toxic agent removal has not been documented. Therefore, the management of this case was unique, as endoscopy with a large working channel duodenoscope was used to remove the mercury from the gastrointestinal tract. Because to the rapid evacuation, blood and urine mercury levels remained low, and systemic toxicity did not develop. We decreased the risk of mercury intoxication to a minimum using this management strategy.
